# Informative Cues Facilitate Saccadic Localization in Blindsight Monkeys

**DOI:** 10.3389/fnsys.2017.00005

**Published:** 2017-02-10

**Authors:** Masatoshi Yoshida, Ziad M. Hafed, Tadashi Isa

**Affiliations:** ^1^Department of System Neuroscience, National Institute for Physiological SciencesOkazaki, Japan; ^2^School of Life Science, The Graduate University for Advanced StudiesHayama, Japan; ^3^Werner Reichardt Centre for Integrative Neuroscience, University of TübingenTübingen, Germany; ^4^Department of Neuroscience, Kyoto University Graduate School of Medicine and Faculty of MedicineKyoto, Japan

**Keywords:** eye movements, covert visual attention, Posner cueing, blindsight, macaque monkeys

## Abstract

Patients with damage to the primary visual cortex (V1) demonstrate residual visual performance during laboratory tasks despite denying having a conscious percept. The mechanisms behind such performance, often called blindsight, are not fully understood, but the use of surgically-induced unilateral V1 lesions in macaque monkeys provides a useful animal model for exploring such mechanisms. For example, V1-lesioned monkeys localize stimuli in a forced-choice condition while at the same time failing to report awareness of identical stimuli in a yes-no detection condition, similar to human patients. Moreover, residual cognitive processes, including saliency-guided eye movements, bottom-up attention with peripheral non-informative cues, and spatial short-term memory, have all been demonstrated in these animals. Here we examined whether post-lesion residual visuomotor processing can be modulated by top-down task knowledge. We tested two V1-lesioned monkeys with a visually guided saccade task in which we provided an informative foveal pre-cue about upcoming target location. Our monkeys fixated while we presented a leftward or rightward arrow (serving as a pre-cue) superimposed on the fixation point (FP). After various cue-target onset asynchronies (CTOAs), a saccadic target (of variable contrast across trials) was presented either in the affected (contra-lesional) or seeing (ipsi-lesional) hemifield. Critically, target location was in the same hemifield that the arrow pre-cue pointed towards in 80% of the trials (valid-cue trials), making the cue highly useful for task performance. In both monkeys, correct saccade reaction times were shorter during valid than invalid trials. Moreover, in one monkey, the ratio of correct saccades towards the affected hemifield was higher during valid than invalid trials. We replicated both reaction time and correct ratio effects in the same monkey using a symbolic color cue. These results suggest that V1-lesion monkeys can use informative cues to localize stimuli in the contra-lesional hemifield, consistent with reports of a human blindsight subject being able to direct attention in cueing paradigms. Because the superior colliculus (SC) may contribute to residual visual capabilities after V1 lesions, and because this structure is important for controlling attentional resources, we hypothesize that our results reflect, among others, SC involvement in integrating top-down task knowledge for guiding orienting behavior.

## Introduction

Blindsight is a phenomenon that occurs in some patients with damage to their primary visual cortex (V1). These patients suffer from a loss of visual awareness in their contra-lesional hemifield, but they are still able to point towards a stimulus when they are forced to guess its location (Weiskrantz, 2009). Blindsight is an intriguing phenomenon for the study of consciousness because it provides a rare occasion in which conscious awareness of salient visual stimuli can be dissociated from other aspects of visual information processing. In addition, blindsight has clinical importance because restoration of visual function, even in the form of blindsight, may improve quality of life in hemianopic patients (Weiskrantz, 2009).

Because of this scientific and clinical importance, development of a blindsight animal model is key to expanding our understanding of this condition. Previous studies have shown that macaque monkeys with a unilateral V1 lesion exhibit residual visual processing as measured by manual key press, reaching, or saccadic eye movements (Humphrey, 1974; Mohler and Wurtz, 1977; Segraves et al., 1987; Cowey and Stoerig, 1995; Yoshida et al., 2008; Schmid et al., 2010). Furthermore, one study (Cowey and Stoerig, 1995) has shown that when asked to report the presence or absence of visual stimuli, V1-lesioned monkeys behaved as if they were unaware of the stimuli. These monkeys thus demonstrated dissociation of visual awareness from forced choice localization, consistent with an objective definition of blindsight. More recently, we have revisited the issue of visual awareness in monkeys with V1 lesions, and using refined behavioral tasks that overcome deficiencies from previous experiments (Yoshida and Isa, 2015). As a consequence, we have identified a behavioral profile in monkeys that resembles blindsight in human subjects who have no visual awareness.

By studying the same monkeys as those used in the study of visual awareness mentioned above (Yoshida and Isa, 2015), we have also shown that: (1) V1-lesioned monkeys are able to maintain the positions of invisible stimuli in their contra-lesional visual field for as long as 2 s (Takaura et al., 2011); (2) gaze during free-viewing is attracted to invisible but visually salient stimuli in the contra-lesional visual field (Yoshida et al., 2012); and (3) non-informative peripheral pre-cues have a facilitatory effect on visually guided saccades to invisible stimuli in the contra-lesional visual field (Ikeda et al., 2011). The remaining question examined in the present study was on whether blindsight monkeys are also able to endogenously orient towards invisible stimuli in the contra-lesional visual field.

Our motivation for exploring endogenous influences on orienting was that a similar question had previously been asked for a human blindsight subject (Kentridge et al., 1999, 2004). Specifically, Kentridge et al. (1999) tested a well-studied blindsight subject (GY) using a Posner cueing task (Posner, 1980), in which an informative cue at the center of the screen (a horizontal arrow) was presented prior to a visual stimulus presented in the subject’s affected hemifield. The pre-cue had a facilitatory effect, meaning that the subject exhibited shorter reaction times for a valid cue than for an invalid cue. These results indicated that the blindsight subject may have been able to pay attention to invisible stimuli in his affected visual field, which has important implications for the contemporary study of consciousness: endogenous attention and conscious awareness are not necessarily one and the same, but they may be distinct entities. Here we asked the same question in blindsight monkeys because such monkeys would confer an unprecedented advantage of exploring, in the near future, neural correlates for both endogenous attention and conscious awareness in a dissociable manner.

In this article, we first show that, in two monkeys with V1 lesions, saccadic localization of visual stimuli in the contra-lesional visual field is facilitated in terms of both correct performance as well as saccadic reaction time when an informative arrow cue on the center of the display is utilized. Then, we supplement these results with data from a variant of the cueing task in which an arrow cue was replaced with a symbolic color cue. Finally, we show that the effects of the pre-cue do not only reflect a bias towards the cued direction, but they also include a putative sensitivity change for detecting saccadic targets in the cued hemifield.

## Materials and Methods

### Subjects

#### Animals

Two Japanese monkeys (Macaca fuscata; monkey A, male, body weight 9.0 kg and monkey T, female, body weight 6.5 kg) were implanted with scleral search coils (Judge et al., 1980) and a head holder. All surgeries were performed under aseptic conditions as described previously (Yoshida et al., 2008). Anesthesia was induced by administration of xylazine hydrochloride (2 mg/kg, i.m.) and ketamine hydrochloride (5 mg/kg, i.m.), and it was maintained with isoflurane (1.0%–1.5%). All experimental procedures were performed in accordance with the recommendations of the National Institutes of Health Guidelines for the Care and Use of Laboratory Animals, and they were approved by the Committee for Animal Experiment at National Institute of Natural Sciences. The monkeys were allowed to recover for more than 2 weeks before starting the preoperative behavioral training.

#### Unilateral V1 Lesion

**Figure 1:**
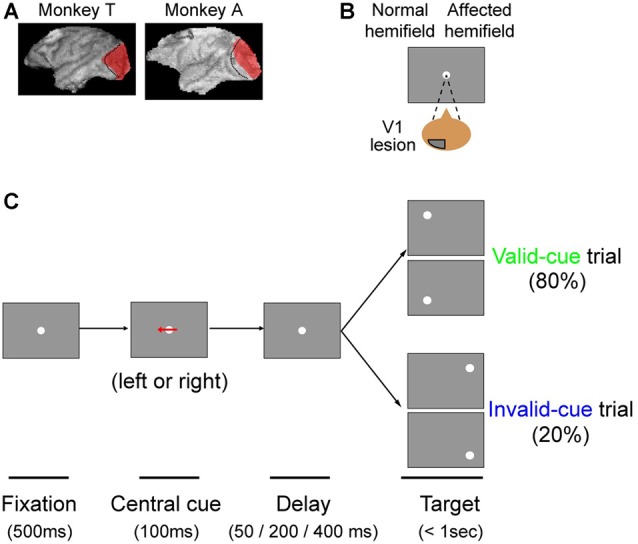
**The arrow cue task. (A)** To illustrate the extent of our primary visual cortex (V1) lesions, 3D images of each monkey’s brain after the lesion procedure were reconstructed from MR images. The lesion site in each animal, estimated from the MR images, is drawn in red. The dotted lines denote the border between V1 and V2. This figure is modified from Figure 1 of Yoshida and Isa (2015), Scientific Reports 5, 10,755. Creative commons (CC BY 4.0). **(B)** Since both monkeys had a lesion in their left V1, their affected hemifield was on the right side of the screen. **(C)** Schematic rectangular screens illustrating the fixation point (FP), central cues and saccadic targets for valid and invalid cue trials. Cues were leftward or rightward arrows. Targets were presented at varying intervals (50, 200 or 400 ms) after the briefly flashed cue (100 ms).

The procedure for making the lesion has been described previously (Yoshida et al., 2008). Briefly, the posterior half of the operculum, the dorsal and ventral leaf and roof of the calcarine sulcus, and the most posterior part of the stem of calcarine sulcus were surgically removed by aspiration with a small-gauge metal suction tube under anesthesia (isoflurane 1.0%–1.5%). After surgery, the monkeys were given penicillin G (80 thousand units/day, i.m.) and cefmetazole (0.5 g/day, i.m.) as antibiotics, as well as dexamethasone sodium phosphate (0.5 mg/kg, i.m.) to minimize brain edema and Diclofenac suppositories for analgesia. The extent of the lesion in each monkey was confirmed as described previously (Yoshida et al., 2008), and is shown in Figure 1A. Briefly, magnetic resonance images (MRIs) were acquired after surgery (Siemens Allegra 3T; MPRAGE-3D; voxel size 0.82 mm × 0.82 mm × 0.81 mm), and they were used to reconstruct a 3D model. Based on the reconstruction and the published literature (Daniel and Whitteridge, 1961; Van Essen et al., 1984), we concluded that the lesion was complete in the relevant area of the contra-lesional visual field used for our behavioral tasks (10° in eccentricity).

### Behavioral Tasks

#### Stimuli

Visual stimuli were presented on a CRT monitor (21 inch, Mitsubishi RD21GZ) positioned 28 cm from the eyes. Visual displays and data storage were controlled using computers running a real-time data acquisition system (Reflective computing, Tempo for Windows) with a dynamic link to Matlab (MathWorks). The CRT monitor was calibrated as described previously (Yoshida et al., 2008). Luminance contrast of the targets was expressed as Michelson Contrast and was varied across trials to draw psychometric curves. The range of luminance contrasts was chosen based on psychometric curves derived from one of our previous studies in the same animals (see Figure 3 in Yoshida et al., 2008). Background luminance was set at 1 or 3 cd/m^2^, because comparable values were chosen in neurophysiological studies that investigated V1 visual responses to stimuli presented in the natural blind spot of macaque monkeys (Murakami et al., 1997; Komatsu et al., 2000).

#### Preoperative Training

The monkeys were placed in a primate chair with their heads fixed, and they were trained to perform a visually guided saccade task with four possible target locations for a liquid reward. Eye movements were recorded using the magnetic search coil (Robinson, 1963), and horizontal and vertical eye positions were sampled at 1 kHz. At the beginning of each trial, a fixation point (FP) appeared at the center of the screen, and the monkeys were required to move their eyes towards it. The FP was a small spot of light of 0.45° in diameter. Fixation duration was varied randomly between 400 ms and 1000 ms, and trials were aborted if eye position deviated by more than 1.5° from the FP during the initial fixation period. After the fixation period, a saccadic target (a small spot of light 0.45° in diameter) appeared in the peripheral visual field concurrently with FP offset. Monkeys were rewarded with fruit juice if saccades were made less than 700 ms after FP offset and if fixation was maintained for 100–300 ms in the target window (size 2–3°). Target eccentricity was fixed at 10°. Target direction was either 30° above or below horizontal for each hemifield. The monkeys were also trained for 1–3 sessions on the main tasks of the current study (see below).

#### Postoperative Training

Postoperative training was started 6 days (monkey A) or 21 days (monkey T) after the lesion surgery, at which time the monkeys’ general behavior in the cage appeared normal. Initial recovery after the V1 lesion was assessed with the visually guided saccade task described in Yoshida et al. (2008). Additionally, a standard procedure to exclude the possibility that light scattering may contribute to residual vision is to test the subject’s ability to detect visual stimuli presented in the natural blind spot of the normal hemifield (Campion et al., 1983; Moore et al., 1995; Gross et al., 2004). We previously confirmed that the monkeys used in this study were not able to use stray light to make correct saccades to stimuli presented in the natural blind spot in the normal, unaffected hemifield (Supplemental Figure 4S of Yoshida et al., 2008).

#### Arrow Cue Task

The task sequence of the present study is illustrated in Figure 1. The task was basically a visually guided saccade task with four possible target locations, as described above. The possible target locations were two in the normal (ipsi-lesional) hemifield and two in the affected (contra-lesional) hemifield (Figure 1A). During an initial fixation period, a horizontal arrow was superimposed on the FP (Figure 1B). The direction of the horizontal arrow (left or right) predicted whether the target would appear in the right or left hemifield with 80% validity; the up/down location of the target was randomly picked. The size of the arrow was 1.7° in width. Targets were presented at varying intervals (50, 200 or 400 ms) after the briefly flashed cue (100 ms). Thus, data for three different cue-target onset asynchronies (CTOAs; 150, 300 and 500 ms) were obtained for valid and invalid cue trials. After the unilateral V1 lesion, monkeys were trained with postoperative training described above and were also tested with other saccade tasks as reported previously. The behavioral tests for the current study were conducted 7 months after the lesion in monkey T (8980 trials in 10 sessions) and 6 months after the lesion in monkey A (9546 trials in eight sessions).

#### Color Cue Task

Monkey T was additionally tested with a color cue task. The task was essentially the same as the arrow cue task, except that the arrow cue was replaced with a color patch. During the initial fixation period, a square patch 3.8° in size was presented for 300 ms with the FP superimposed on it. A magenta patch predicted left targets with 80% validity, whereas a green patch predicted right targets with 80% validity. Targets were presented at varying intervals (50, 200 or 400 ms) after the briefly flashed cue. Thus, data for three different CTOAs (350, 500 and 700 ms) were obtained. In order to familiarize the monkey with the contingencies between color cues and target locations, we first trained it in separate sessions with 100% valid cues intermixed with no-cue trials, before we eventually ran our current experiments. The behavioral tests presented in this article for this color cue task were conducted at 8–9 months after the lesion in monkey T, and also after the sessions with the arrow cue task described above (7011 trials in nine sessions).

Note that during preoperative training, we found that Monkey A failed to convincingly demonstrate successful association of the color cue with the hemifield that it predicted. Thus, we dropped Monkey A from further testing with the color cue task after the lesion.

### Data Analysis

#### Analysis of Saccadic Eye Movements

Calibration procedures for saccade detection have been described previously (Aizawa and Wurtz, 1998). Target localization was evaluated by calculating the ratio of success trials among all trials (“proportion correct”). A trial was considered successful when the monkeys made a saccade to the quadrant containing the target. Since the monkeys were trained to make accurate saccades as described previously (Yoshida et al., 2008), directional errors for correct saccades were less than 15°. Also, since there were four possible target locations, chance performance would have been 25% correct. We also measured saccadic reaction time, defined as the interval between saccade and target onset. Saccades were initially identified based on peak velocity of the polar component of eye data exceeding 100°/s. Then, the onset time of the detected saccade was defined as the time point preceding the detected peak-time at which the velocity exceeded 100°/s. Trials in which monkeys broke fixation before FP offset (see above) were discarded. Also, there was a small number of trials with anticipatory saccades, defined here as trials with <70 ms saccadic reaction time; these trials were excluded from analysis (<0.1% in total trials in both monkeys).

#### Analysis of Saccadic Reaction Time and Fitting of Psychometric Curves

All of the analyses were conducted using Matlab 2016b (Mathworks). For statistical analysis of saccadic reaction times, Wilcoxon’s ranksum test with Bonferroni correction for multiple comparisons was used to compare valid and invalid cue trials. As part of our experiment, we varied the luminance contrast of the target. This allowed us to obtain psychometric curves of sensitivity to luminance contrast. For fitting of psychometric curves, psignifit 4 (Schütt et al., 2016) was used. Data were fitted with cumulative Gaussian distribution function, and the parameters were determined from maximum a posteriori (MAP) estimates using the maximum likelihood method. For comparison of thresholds of psychometric curves for valid and invalid cue trials, permutation tests were used; randomly sampled data were generated from pooled data with both valid and invalid cue trials. Then, differences between thresholds for resampled valid and invalid cue trials were calculated. This procedure was repeated 9999 times to build a distribution of the null hypothesis that the data for valid and invalid cue trials were extracted from the same population. *P*-values were calculated by comparing the distribution and the experimental data.

## Results

### Training, Lesion and Recovery

We trained two Japanese macaque monkeys on a visually guided saccade task before surgically inducing a unilateral V1 lesion. Both monkeys attained >95% proportion correct, after which we surgically removed the left V1 (Figure 1A; see “Materials and Methods” Section). We assessed the lesion extent as described previously (Yoshida et al., 2008; also see “Materials and Methods” Section). Briefly, using a visually guided saccade task with a five-alternative forced choice condition, we confirmed previously that the threshold for luminance contrast was significantly increased in the contra-lesional affected visual field (Yoshida et al., 2008). However, even though the proportion correct for a visually guided saccade task with two alternative forced choices decreased to near chance levels just after the lesion, it recovered to >90% and became stable at approximately 8 weeks after the lesion (Yoshida et al., 2008). Thus, the monkeys were in an ideal position to perform the endogenous cueing paradigms of the present article.

### Arrow Cue Task

In this study, we tested our two monkeys with an arrow cue task (Figures 1B,C). The task was basically a visually guided saccade task with four possible target locations. The possible target locations were two in the normal (ipsi-lesional) hemifield and two in the affected (contra-lesional) hemifield (Figure 1B). During an initial fixation period, a horizontal arrow was superimposed on the FP (Figure 1C; see “Materials and Methods” Section). To evaluate the effect of the central pre-cue on saccadic localization, we analyzed both proportion correct and saccadic reaction time.

**Figure 2:**
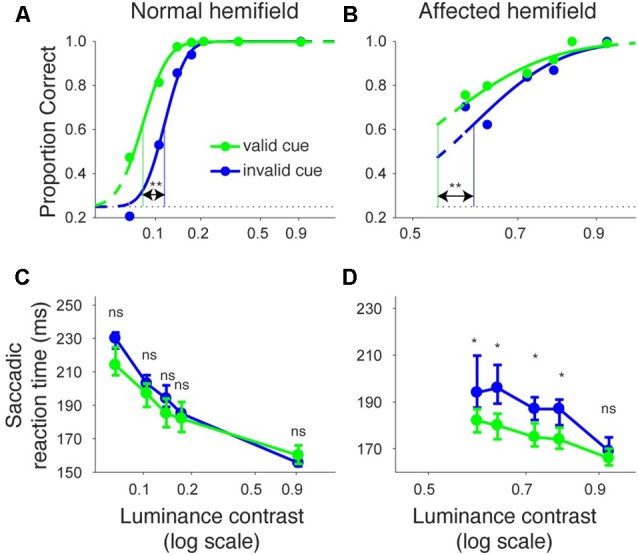
**Psychometric curves and saccadic reaction times for the arrow cue task (monkey T). (A,B)** Dots indicate proportion correct at various luminance contrasts. Data were fitted by psychometric curves (lines). The dots and lines are shown in green for valid cue trials and in blue for invalid cue trial. Horizontal lines indicate chance level performance (0.25 for four alternative forced choice tasks). Vertical lines indicate thresholds for each condition. The threshold was defined as the luminance contrast at which a psychometric curve crossed a value of 0.625 (=(1 + 0.25)/2). **Significantly different (*p* < 0.01; permutation test). **(C,D)** Dots indicate median saccadic reaction time at various luminance contrasts. Error bars indicate the 40th and 60th percentiles of the data. Asterisks (*p* < 0.05) and ns (not significant) indicate results of Wilcoxon’s ranksum test with Bonferroni correction for multiple comparisons. Only data points with more than 10 correct trials were displayed. For both rows, the left column shows data for trials with targets presented in the normal (ipsi-lesional) hemifield **(A,C)**, and the right column shows data for trials with targets presented in the affected (contra-lesional) hemifield **(B,D)**.

Figures 2A,B shows the proportion of correct trials across different luminance contrasts of the target for monkey T. When the target was presented in the normal hemifield (Figure 2A), the proportion of correct trials became lower and almost at chance level (0.25) when the luminance contrast became lower, regardless of cue validity. This typical pattern of saccadic localization can be fitted with a psychometric curve in the form of a cumulative Gaussian function. When the target in the left, normal hemifield was preceded by a valid cue (i.e., a leftward arrow), the psychometric curve (green line) was shifted leftward relative to the curve (blue) obtained when the cue was invalid (i.e., a rightward arrow). This indicates that the cue affected task performance, as might be expected from an intact animal. We quantitatively evaluated the shift of the psychometric curve associated with cue validity. We defined the threshold for the psychometric curve as the luminance contrast at which the psychometric curve crossed a proportion correct value of 0.625 (=(1 + 0.25)/2). In the normal hemifield, thresholds for the valid and invalid cue trials were 0.08 and 0.12, respectively, and the difference between these thresholds was statistically significant (*p* < 0.0001; permutation test). When the target was presented in the right, affected hemifield (Figure 2B), the overall thresholds were higher than those for the normal hemifield (compare x-axes between affected and normal hemifield curves). This is evidence that the V1 lesion really did affect visual information processing (Yoshida et al., 2008). However, the presence of psychometric curves at all suggests that the V1-lesioned monkeys did indeed exhibit blindsight (Yoshida and Isa, 2015). In any case, when we now compared thresholds for valid and invalid cue trials, we found that they were 0.58 and 0.63, respectively. The difference between these thresholds was statistically significant (*p* = 0.0037; permutation test). These results indicate that informative, central pre-cues can improve performance in saccadic localization.

We also examined saccadic reaction times during the same task (Figures 2C,D). When median reaction time for targets in the left, normal hemifield was plotted across various target contrasts (Figure 2C), we found that reaction time during valid trials was similar to reaction time during invalid trials. Wilcoxon’s ranksum test with Bonferroni correction for multiple comparisons showed that saccadic reaction time at each luminance contrast was not significantly different between the valid and invalid cue trials (“ns” in Figure 2C). As for the affected hemifield, pre-cues showed a strong benefit in valid cue trials. The same statistical test revealed that reaction time at each luminance contrast was significantly shorter in the valid cue trials than in the invalid cue trials (*p* < 0.05; * in Figure 2D), except for targets with the highest luminance contrast. These results indicate that the central, pre-cue had a facilitatory effect on saccadic localization both for the normal and affected hemifields in monkey T.

**Figure 3:**
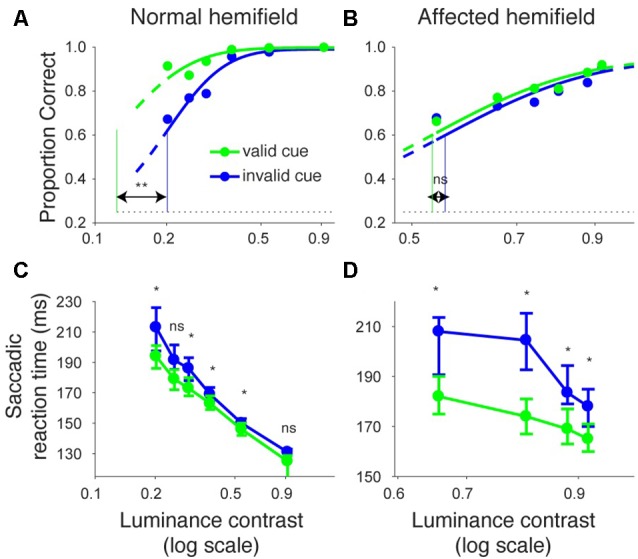
**Psychometric curves and saccadic reaction times for the arrow cue task (monkey A).** This figure uses the same formatting as Figure 2 but shows data for monkey A.

We repeated the same analyses for monkey A (Figure 3). Figures 3A,B shows the proportion of correct trials across different target contrasts. In the normal hemifield (Figure 3A), thresholds for the valid and invalid cue trials were 0.15 and 0.21, respectively, and the difference between these thresholds was statistically significant (*p* < 0.0001; permutation test). Thus, the valid cue improved performance in the normal hemifield. When the target was presented in the right, affected hemifield (Figure 3B), thresholds for valid and invalid cue trials were 0.62 and 0.64, respectively. Despite the tendency for a lower threshold on valid trials, the difference between the two thresholds was not significant in this animal (*p* = 0.36; permutation test). However, examining saccadic reaction times (Figures 3C,D), we still found a strong effect of cueing in the affected hemifield (Figure 3D). Specifically, when median reaction time for targets in the left, normal hemifield was plotted across various target contrasts (Figure 3C), we found that reaction time during valid trials was shorter than reaction time during invalid trials. Wilcoxon’s ranksum test with Bonferroni correction for multiple comparisons showed that reaction times at some luminance contrasts were significantly shorter in the valid cue trials than in the invalid cue trials (*p* < 0.05; * in Figure 3C). As for the affected hemifield, the monkey also showed a cueing benefit. The same statistical test showed that reaction time at each luminance contrast was significantly shorter in the valid cue trials than in the invalid cue trials (*p* < 0.05; * in Figure 3D). These results indicate that the central, pre-cue had a facilitatory effect on saccadic localization both for the normal and affected hemifields in monkey A, and they also confirm that monkey A still benefited from the cue despite the mild psychometric curve effect in Figure 3B.

**Figure 4:**
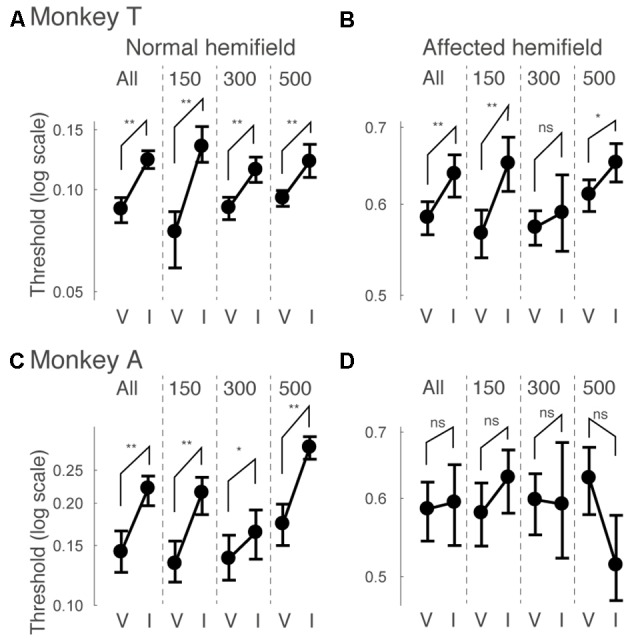
**Thresholds for different cue-target onset asynchronies (CTOAs) in the arrow cue task.** Thresholds defined for psychometric curves (see the legend of Figure 2 and texts) were compared between valid cue trials (“V”) and invalid cue trials (“I”) for monkey T **(A,B)** and for monkey A **(C,D)**. Error bars indicate 68% (=1SD) confidence intervals for the thresholds. Four comparisons were plotted in one figure: “All” for data from all CTOAs combined, “150” for data with 150 ms CTOA, “300” for data with 300 ms CTOA and “500” for data with 500 ms CTOA. The left column shows data for trials with targets presented in the normal (ipsi-lesional) hemifield **(A,C)**. The right column shows data for trials with targets presented in the affected (contra-lesional) hemifield **(B,D)**. **p < 0.01, *p < 0.05 and ns (not significant) indicate results of permutation tests for the difference between thresholds for valid and invalid cue trials.

Next, we examined how the facilitatory effect of the central arrow cue on task performance was modulated as a function of CTOAs. We calculated psychometric curve thresholds (see for example Figure 2 and the corresponding text) not only for data from all CTOAs combined (Figures 2, 3), but also separately for 150, 300 and 500 ms CTOAs. These thresholds are shown in Figure 4 for valid cue trials (“V”) and invalid cue trials (“I”). As can be seen, in the normal hemifield (Figures 4A,C), differences in threshold between valid and invalid trials were highest for the shortest CTOA (150 ms) for both monkeys. However, each CTOA showed a cueing effect, as assessed by permutation tests for the difference between thresholds for valid and invalid cue trials (asterisks in Figures 4A,C). In the affected hemifield, a similar tendency was visible in monkey T (Figure 4B) and in monkey A (Figure 4D). Permutation tests for the difference between thresholds for the valid and invalid cue trials showed significant differences in monkey T but not in monkey A (asterisks in Figures 4B,D).

**Figure 5:**
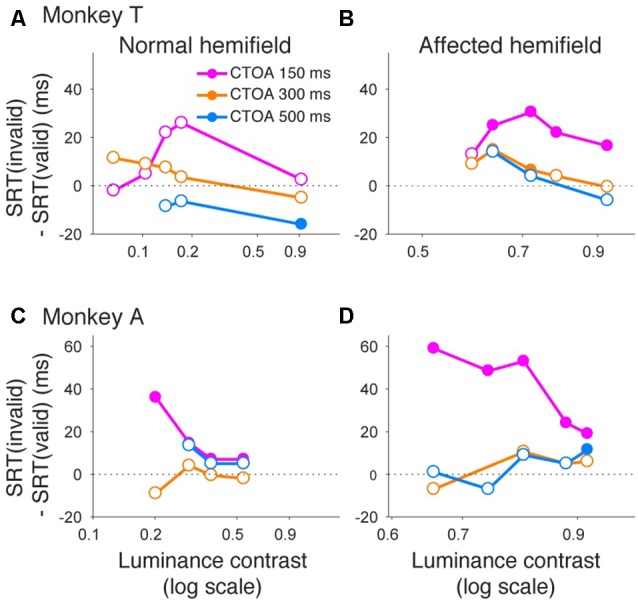
**Saccadic reaction times for different CTOAs in the arrow cue task.** Differences between median reaction time for invalid cue trials and median reaction time for valid cue trials were plotted across luminance contrasts for monkey T **(A,B)** and for monkey A **(C,D)**. Data for the normal hemifield **(A,C)** and for the affected hemifield **(B,D)** are separately displayed. Colors of the plot denote data for different CTOAs (magenta, 150 ms; orange, 300 ms; light blue, 500 ms). Filled circles indicate statistically significant differences from zero, and open circles indicate non-significant differences (Wilcoxon’s ranksum test with Bonferroni correction for multiple comparisons). In the affected hemifield, both monkeys showed reaction time benefits after pre-cueing, especially in the shortest CTOA. There was also no cost associated with longer CTOAs, as might be expected from inhibition of return (IOR).

Similar to what we did for psychometric curve thresholds, we also examined how the facilitatory effect of the central arrow cue on saccadic reaction time was modulated as a function of CTOA. We calculated differences between median reaction time for invalid cue trials and median reaction time for valid cue trials, but this time as a function of both luminance contrast and CTOA. Positive values indicate attentional benefits and negative values indicate so-called inhibition-of-return (IOR) effects. In the normal hemifield (Figures 5A,C), differences in median reaction time between valid and invalid trials were generally small. However, Wilcoxon’s ranksum test with Bonferroni correction for multiple comparisons detected IOR at the longest CTOA (500 ms) in monkey T (filled circles in Figure 5A) and a facilitatory effect at the shortest CTOA (150 ms) in monkey A (filled circles in Figure 5C). In the affected hemifield (Figures 5B,D), differences in median reaction time between valid and invalid trials were highest at the shortest CTOA (150 ms) for both monkeys (Wilcoxon’s ranksum test with Bonferroni correction for multiple comparisons; *p* < 0.05). Taken together, analysis of performance and saccadic reaction time for each CTOA (Figures 4, 5) suggests that the facilitatory effects of pre-cues in the affected hemifield were highest for the shortest CTOA.

### Color Cue Task

**Figure 6:**
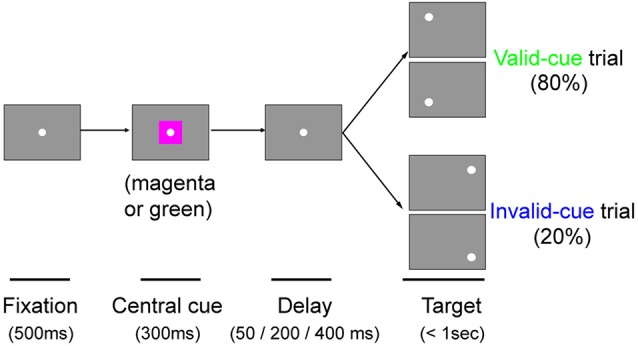
**The color cue task.** Schematic rectangular screens illustrating the FP, central cue and saccadic targets for valid and invalid cue trials. Cues were square patches. A magenta patch predicted left targets with 80% validity. A green patch predicted right targets with 80% validity. Targets were presented at varying intervals (50, 200 or 400 ms) after the briefly flashed cue (300 ms).

**Figure 7:**
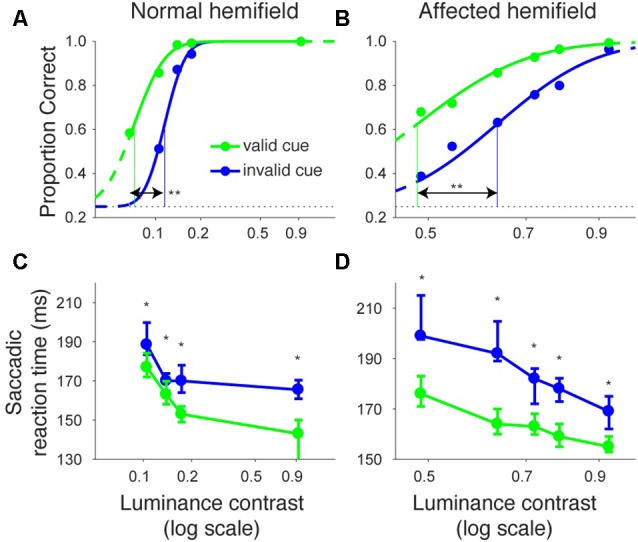
**Psychometric curves and saccadic reaction times for the color cue task (monkey T).** This figure is formatted similarly to Figure 2, but shows data for the color cue task in monkey T.

Even though the arrow cue was presented at the center of the screen, it is not purely symbolic but has a spatial component. Specifically, the asymmetry in the shape of the arrow could have biased performance from a purely sensory stimulus-response association. Thus, to examine the effects of purely symbolic pre-cues, we designed another task in which we used a color patch as the pre-cue. We tested one of the monkeys (monkey T) with the color cue task to support the conclusions obtained above from the arrow cue task. The task was essentially the same as the arrow cue task, but the arrow cue was replaced with a color patch (Figure 6). When the target presented in the left, normal hemifield was preceded by a valid color cue (Figure 7A), the psychometric curve (green line) was shifted leftward from the psychometric curve when the left target was preceded by an invalid color cue, similar to what we observed with the same monkey using the arrow cue. In the current experiment, thresholds for the valid and invalid cue trials were 0.07 and 0.11, respectively, and the difference was statistically significant (*p* < 0.0001; permutation test). Importantly, when the target was presented in the right, affected hemifield (Figure 7B), the thresholds for valid and invalid cue trials were 0.47 and 0.62, and, once again, the difference between them was statistically significant (*p* < 0.0001; permutation test). These results indicate that the monkey was able to use the symbolic color cue to improve performance in saccadic target localization.

We also examined reaction times in the color cueing task (Figures 7C,D). When median saccadic reaction time for targets in the left, normal hemifield was plotted across various luminance contrasts, reaction time was shorter during valid than during invalid cue trials (Figure 7C). Wilcoxon’s ranksum test with Bonferroni correction for multiple comparisons showed that reaction time at each luminance contrast was generally shorter in valid than in invalid cue trials (*p* < 0.05; asterisks in Figure 7C). For the affected hemifield, median saccadic reaction time was also shorter in the valid cue trials (Figure 7D): the same statistical test showed that reaction time at each luminance contrast was shorter in valid than in invalid cue trials (*p* < 0.05; asterisks in Figure 7D). These results indicate that the central, color cue had a facilitatory effect on saccadic localization both for the normal and affected hemifields.

**Figure 8:**
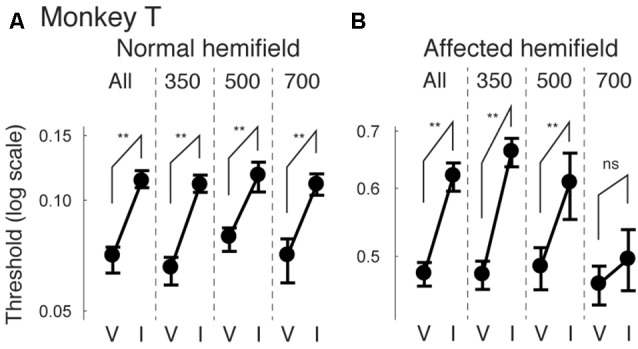
**Thresholds for different CTOAs in the color cue task.** Thresholds defined for the psychometric curves of Figures 7A,B were compared between valid cue trials (“V”) and invalid cue trials (“I”) for monkey T **(A,B)**. This figure follows the same conventions as those in Figure 4.

**Figure 9:**
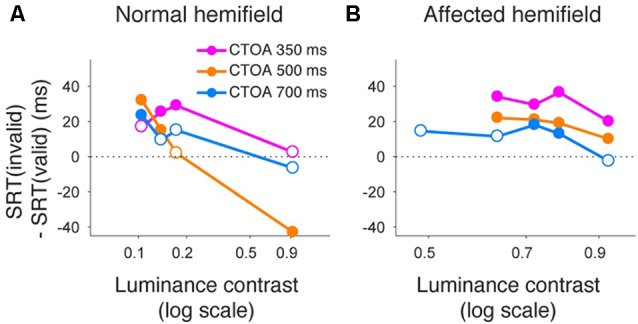
**Saccadic reaction times for different CTOAs in the color cue task.** Differences between median reaction time for invalid cue trials and median reaction time for valid cue trials were plotted across luminance contrasts for monkey T. This figure follows the same conventions as those in Figure 5. Similar to the arrow cue task, pre-cueing using color symbols in the affected hemifield was again associated with a benefit in reaction time, especially for the shortest CTOA.

Finally, we also separated color cueing trials based on CTOAs (Figures 8, 9). When psychometric curve thresholds were compared between valid (“V”) and invalid (“I”) trials, differences were highest for the shortest CTOA (350 ms) for both the normal (Figure 8A) and affected (Figure 8B) hemifields. Permutation tests for the difference between thresholds for valid and invalid cue trials showed highly significant differences in both hemifields (asterisks in Figures 8A,B), except for trials with 700 ms CTOA in the affected hemifield. Similarly, when differences between median reaction times for valid and invalid trials were plotted across luminance contrasts (Figure 9), differences were highest for the shortest CTOA (350 ms), especially in the affected (Figure 9B) hemifield (Wilcoxon’s ranksum test with Bonferroni correction for multiple comparisons; *p* < 0.05). Taken together, analysis of performance and saccadic reaction times for each CTOA individually (Figures 8, 9) suggests that the facilitatory effects of pre-cueing in this task were highest for the shortest CTOA.

### Sensitivity vs. Bias

One of the problems associated with a standard pre-cueing attention task (a left or right arrow cue with a left or right target) is that attention may be confounded by reward expectation in animal studies (Maunsell, 2004). This is because information in the pre-cue can directly bias the subject’s reward expectation towards the target stimulus rather than facilitate sensory processing *per se*. For example, even if a monkey was not able to detect the cued target, the monkey might learn the contingency between the cue (80% valid) and the rewarded hemifield. Here, we call this distinction a “sensitivity vs. bias issue”.

The experimental paradigm adopted in our study had an advantage to potentially help overcome this issue. Specifically, since the task was a four-alternative forced choice task (rather than the standard two-alternative forced choice task), our pre-cues only provided partial information about target location (i.e., which hemifield it would appear in, but not whether it was in the upper or lower visual field). For example, if a rightward cue was presented, there was still uncertainty about whether the target could appear in the lower right or upper right location. Thus, we could dissociate the effect of bias (left hemifield or right hemifield based on the cue) from the effect of pure sensitivity improvement for target detection (in which the cue might boost sensitivity in the cued visual hemifield). In our task, the proportion correct during the pre-cue tasks can be decomposed into two components. The “bias” component was evaluated by the proportion correct for the left-vs.-right location of the target, irrespective of the up-down location (“LR correct”). In other words, we measured proportion correct based on rightward or leftward saccade direction, independent of whether the monkey made a correct saccade to the up/down target location. On the other hand, the “sensitivity” component was evaluated by the proportion correct for the up-down location, irrespective of the left-right location (“UD correct”).

**Figure 10:**
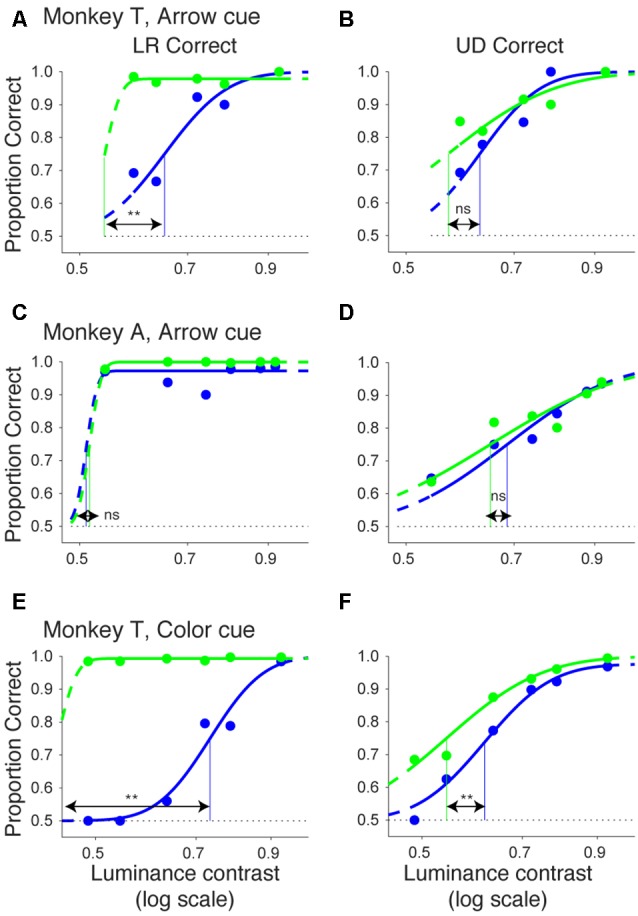
**Bias vs. sensitivity for psychometric curves in the arrow and color cue tasks.** As variants of psychometric curves, two different kinds of proportion correct were calculated and plotted across luminance contrasts for the arrow cue task in monkey T **(A,B)**, for the arrow cue task in monkey A **(C,D)**, and for the color cue task in monkey T **(E,F)**. In the left column **(A,C,E)**, proportion correct for left-right choice irrespective of up-down choice was calculated (“LR correct”). In the right column **(B,D,F)**, proportion correct for up-down choice irrespective of left-right choice was calculated (“UD correct”). The data were fitted by cumulative Gaussian functions (lines). The dots and lines are shown in green for valid cue trials and in blue for invalid cue trial. Horizontal lines indicate chance level performance (0.5 for two alternative forced choice tasks). Vertical lines indicate thresholds for each condition. The threshold was defined as the luminance contrast at which a psychometric curve crossed a value of 0.75 (=(1 + 0.5)/2).

**Figure 11:**
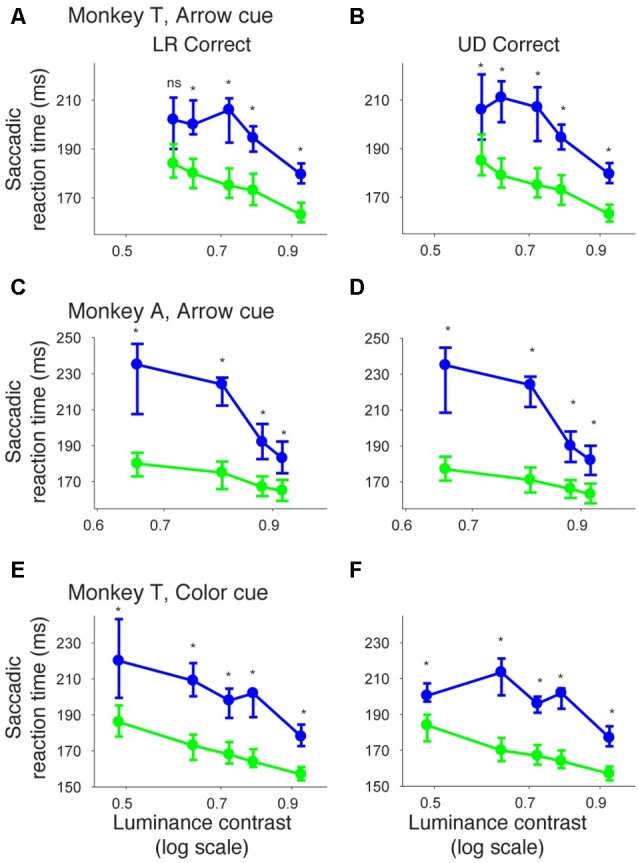
**Bias vs. sensitivity for saccadic reaction times in the arrow and color cue tasks.** Median reaction time for “LR correct” trials (the left column) and “UD correct” trials (the right column) were plotted across luminance contrasts for the arrow cue task in monkey T **(A,B)**, for the arrow cue task in monkey A **(C,D)**, and for the color cue task in monkey T **(E,F)**. The dots and lines are shown in green for valid cue trials and in blue for invalid cue trial. Error bars denote the 40th and 60th percentiles of the data.

In Figure 10, we plotted psychometric curves for “LR correct” and “UD correct” for trials with targets in the affected hemifield. Since the effects on threshold were highest when the CTOA was shortest (Figures 4, 5, 8, 9), we plotted the data with the shortest CTOA (150 ms for the arrow cue task and 350 ms for the color cue task). For both monkeys, trials with a valid cue had higher “LR correct” proportion than those with an invalid cue (Figures 10A,C,E). These results suggest that the monkeys used the information of the direction of the arrow cue to direct their gaze to either left or right hemifields. For both monkeys, trials with the valid cue had lower threshold for “UD correct” (Figures 10B,D,F) than those with the invalid cue (0.56 vs. 0.61 in monkey T and 0.60 vs. 0.64 in monkey A for the arrow cue task and 0.53 vs. 0.60 in monkey T for the color cue task). However, permutation tests showed that these differences were not always significantly different: *p* = 0.12 in monkey T and *p* = 0.23 in monkey A for the arrow cue task; *p* < 0.001 in monkey T for the color cue task. We thus also checked reaction times for “LR correct” and “UD correct” saccades (Figure 11). For both monkeys, “LR correct” trials with a valid cue had shorter reaction times than those with an invalid cue (*p* < 0.05, Wilcoxon’s ranksum test with Bonferroni correction; Figures 11A,C,E). Perhaps more interestingly, for both monkeys, “UD correct” trials with a valid cue also had shorter reaction times than those with an invalid cue (*p* < 0.05, Wilcoxon’s ranksum test with Bonferroni correction; Figures 11B,D,F). These results suggest that the monkeys not only biased their choice to the hemifield that the pre-cue indicated, but they also tended to direct their attention to the affected hemifield, thus facilitating detection of the saccadic target.

## Discussion

In this article, we first showed that, in two monkeys with V1 lesions, saccadic localization of visual stimuli in the contra-lesional visual field was facilitated by an arrow pre-cue in terms of both correct performance and saccadic reaction time (Figures 2–5). Next, these results were supplemented in one monkey with data from a variant of a Posner task in which an arrow cue was replaced with a symbolic color cue (Figures 7–9). Finally, we showed that the effects of a pre-cue were not necessarily only restricted to bias towards the cued direction, but may have also involved sensitivity changes by facilitating detection of the saccadic target either in terms of accuracy and/or reaction time in the cued direction (Figures 10, 11). Our results suggest that monkeys with a unilateral V1 lesion are able to use informative cues in a top-down manner to process stimuli in the contra-lesional hemifield. Since we used the identical stimulus set in which the same monkeys had previously failed to report awareness (Yoshida and Isa, 2015), these results suggest that the monkeys were able to direct top-down resources to invisible stimuli. These results are consistent with findings in a human blindsight subject who was able to direct attention in a Posner paradigm (Kentridge et al., 1999, 2004).

In the current study, analysis of different CTOAs (Figures 4, 5, 8, 9) revealed that the shortest CTOA (150 ms for the arrow cue task and 350 ms for the color cue task) had the strongest facilitatory effects. It is interesting to compare this observation with one of our previous studies, in which we tested V1-lesioned monkeys with saccade tasks using non-informative peripheral pre-cues (Ikeda et al., 2011). In that previous study, the facilitatory effect on saccadic reaction time was highest at 100 ms CTOA for both hemifields. These findings, coupled with ours in the current study, are consistent with human studies in which the effects of central cues generally have a slower time course than those of peripheral cues (e.g., Nakayama and Mackeben, 1989; Cheal and Lyon, 1991). Another point of note is that in the current study, there was no case for statistically significant IOR effects in the affected hemifield (Figures 5B,D, 9B). This is consistent with our previous study using non-informative peripheral pre-cues (Ikeda et al., 2011) and further suggests impairment of IOR after V1 lesions, even when endogenous attention is employed.

Our result showed consistent facilitatory effects of pre-cues in our two monkeys. However, the individual effects associated with such facilitation were slightly different from individual to individual; monkey T showed relatively larger effects on proportion correct (Figure 1), whereas monkey A showed relatively larger effects on saccadic reaction time (Figure 2). This difference can be explained by individual differences in speed-accuracy tradeoffs (Heitz, 2014) and may arise because the tasks used in the current study were a class of reaction time tasks (in which subjects are able to respond to the target as soon as possible). It would be interesting in future studies to investigate whether central cues facilitate performance when monkeys are tested with another class of discrimination tasks in which subjects have to wait for a fixed duration before responding to the target.

To study exogenous, overt attention in monkeys, informative peripheral cues have been used as a variant of the Posner task in many laboratories (Bowman et al., 1993; Voytko et al., 1994; Witte et al., 1996; Ignashchenkova et al., 2003; Bell and Munoz, 2008; Monosov and Thompson, 2009). In these studies, involvement of parietal cortex (Robinson et al., 1995) and superior colliculus (SC; Robinson et al., 1995) has been suggested. For blindsight monkeys, our group previously demonstrated that non-informative peripheral cues had facilitatory effects on saccadic reaction time (Ikeda et al., 2011). To our knowledge, our study is the first demonstration of endogenous attention using Posner paradigms with informative central pre-cues in (not only blindsight but also normal) macaque monkeys.

In human psychophysics, it is already known that endogenous attention cued by an informative peripheral cue shifts the psychometric curve of contrast sensitivity leftward, thereby supporting the idea that attention may enhance sensory signals (Cameron et al., 2002). Our results suggest that such enhancement of sensory signals can be done without V1. Then, how might endogenous attention be mediated? Previously, we showed that the SC showed sustained activity during a spatial memory task (Takaura et al., 2011), and we argued for a possible contribution of top-down signals from prefrontal cortex (Johnston and Everling, 2006) in maintaining sustained SC activity. This kind of top-down signal may facilitate cortical and subcortical attentional networks, which are composed of the dorsal cortical visual pathway, the ventral cortical visual pathway, the prefrontal cortex, pulvinar, SC and so on (Veale et al., 2017).

Our results also have implications that may impact contemporary consciousness research. As already explained earlier, Kentridge et al. (1999) showed that a well-studied blindsight subject GY was able to pay attention to invisible stimuli in his affected visual field. The authors concluded that endogenous attention and conscious awareness are not one and the same, but they may be different entities. Our study provides consistent results in blindsight monkeys, thereby contributing to accumulating evidence of striking similarities between behaviors (and possibly subjective experiences, too) of blindsight humans and monkeys. Our findings open the possibility to reveal neural correlates for endogenous attention and for conscious awareness separately, using neurophysiological approaches, as a next step.

Another direction that will be of interest is to build a computational model of attention and decision making based on these findings. Previously, we used a diffusion model, a class of evidence-accumulation models, to fit model parameters and to explain localization performance as well as the distribution of saccadic reaction time in a visually guided saccade task (Yoshida et al., 2008). These analyses revealed that the decision threshold in blindsight monkeys is reduced. In other words, blindsight monkeys become less deliberate after V1 lesions. Given our present results, the next question will be on how the pre-cue affects threshold and sensitivity in decision processes during our four alternative forced choice task adopted in the current study. Moreover, another important clue that can give interesting insights about sub-threshold decision processes during pre-cue and saccade tasks is the pattern of microsaccades that our monkeys generated. It is already known that the number and direction of microsaccades are affected by covert attention (Hafed and Clark, 2002). In turn, we can read out sub-threshold decision process from the frequency and direction of microsaccades (Tian et al., 2016). Such analysis will not only demonstrate the potential role of V1 and SC in the patterns of microsaccades, but it will also contribute to building an integrated computational model of attention, decision and eye movements (Hafed et al., 2015).

## Author Contributions

MY designed the experiments and collected the behavioral data; MY and TI performed the surgeries; MY and ZMH analyzed the data; MY, TI and ZMH wrote and edited the final draft.

## Conflict of Interest Statement

The authors declare that the research was conducted in the absence of any commercial or financial relationships that could be construed as a potential conflict of interest. The reviewer YK declared a shared affiliation, though no other collaboration, with one of the authors TI to the handling Editor, who ensured that the process nevertheless met the standards of a fair and objective review.
